# Dissolvable Calcium Alginate Microfibers Produced via Immersed Microfluidic Spinning

**DOI:** 10.3390/mi14020318

**Published:** 2023-01-26

**Authors:** Tuo Zhou, Sahar NajafiKhoshnoo, Rahim Esfandyarpour, Lawrence Kulinsky

**Affiliations:** 1 Mechanical and Aerospace Engineering, University of California Irvine, 5200 Engineering Hall, Irvine, CA 92627, USA; 2 Materials and Manufacturing Technology, University of California Irvine, 5200 Engineering Hall, Irvine, CA 92627, USA; 3 Electrical Engineering and Computer Science, University of California Irvine, 5200 Engineering Hall, Irvine, CA 92627, USA; 4 Biomedical Engineering, University of California Irvine, 5200 Engineering Hall, Irvine, CA 92627, USA

**Keywords:** immersed microfluidic spinning, microfibers, tissue engineering, biofabrication

## Abstract

Fabrication of micro- and nanofibers are critical for a wide range of applications from microelectronics to biotechnology. Alginate microfibers with diameters of tens to hundreds of microns play an important role in tissue engineering and fibers of these diameters are impossible to fabricate via electrospinning and can only be produced via fluidic spinning. Typically, microfluidic spinning based on photopolymerization produces fibers that are not easily dissolvable, while fluidic spinning with chemical cross-linking employs complex setups of microfabricated chips or coaxial needles, aimed at precise control of the fiber diameter; however, fluidic spinning introduces significant cost and complexity to the microfluidic setup. We demonstrate immersed microfluidic spinning where a calcium alginate microfiber is produced via displacement of alginate solution through a single needle that is immersed in a cross-linking bath of calcium chloride solution. The resulting diameter of the fiber is characterized and the fiber diameter and topology of the deposited fiber is related to the concentration of the alginate solution (2 wt%, 4 wt%, and 6 wt%), needle gauge (30 g, 25 g, and 20 g), and the volumetric flow rate of the alginate solution (1 mL/min, 2 mL/min, and 2.7 mL/min). The resulting fiber diameter is smaller than the internal diameter of the needle and this dependence is explained by the continuity of the flow and increased rate of fall of the liquid jet upon its issuing from the needle. The fiber diameter (demonstrated diameter of fibers range from 100 microns to 1 mm) depends weakly on the volumetric flow rate and depends strongly on the needle diameter. It also seems that for a smaller needle size, a greater concentration of alginate results in smaller diameter fibers and that this trend is not evident as the needle diameter is increased. In terms of topology of the deposited fiber, the higher wt% alginate fiber produces larger loops, while smaller wt% alginate solution yields a denser topology of the overlaid fiber loops. These fibers can be dissolved in DMEM/EDTA/DSC solution in 20–30 min (depending on the fiber diameter), leaving behind the hollow channels in the hydrogel matrix. We believe that the demonstrated simple setup of the immersed microfluidic spinning of the calcium alginate microfibers will be useful for creating tissue constructs, including the vascularized tissue implants.

## 1. Introduction

Micro- and nanofibers characterized by high surface area, predictable geometry, long aspect ratio, and high speed of production have important applications in a wide range of fields from composite materials and wearable electronics to water collection and tissue engineering [1]. In tissue engineering, one of the critical problems is that of tissue vascularization, i.e., creating a network of fluidic channels of high density for delivery of nutrients to the cells of the bioengineered tissue in the period from days to weeks (depending on the size of the implanted tissue) while the body’s own blood vessels are developing within a tissue construct [2].

One of the approaches for the fabricated microfluidic network within a tissue construct can be the creation of the sacrificial network of microfibers inside the cell-laden gel matrix. In cases when the fiber network is made from natural carbohydrate alginate that can be dissolved in ethylenediaminetetraacetic acid (EDTA), the dissolution of the alginate fibers would leave behind hollow microfluidic channels suitable for the transport of nutrients to the cells of the tissue.

Out of two main methods for the fabrication of micro and nanochannels—electrospinning and fluidic spinning—only fluidic spinning is capable of producing fibers with a diameter larger than several microns [3]. In order to utilize the fast transport of nutrients to the cells and to enable fast fiber dissolution, fibers with diameters in the range between several microns and dozens of microns are necessary. Therefore, we focused on using fluidic spinning for the production of alginate microfibers.

A recent review [4] surveys various manufacturing technologies for the production of calcium alginate fibers including freeze-drying [5,6], wet-spinning technology [7,8,9], an immersive centrifugal jet spinning technique [10,11], and microfluidic spinning technology [12,13,14,15]. Freeze-drying is, by itself, not a method of producing the fibers, but rather a post-fabrication technique to introduce porosity within alginate fibers during the freeze-drying stage once fibers are produced.

The fluidic spinning of microfibers involves the flow of liquid solution in a microchannel and that solution is solidified upon either exposure to a chemical cross-linker or via UV light acting on a photoinitiator within the solution (i.e., via photopolymerization) [12]. Typically, photopolymerized material is more difficult to dissolve, and thus the photopolymerization technique is less suitable for the fabrication of sacrificial microfiber networks; therefore, our present work focuses on studying the chemically cross-linkable system of alginate that is solidified upon exposure to CaCl_2_ solution to produce calcium alginate microfibers.

In wet spinning technology, the alginate fibers ejected via spinneret are drawn through the chemical cross-linking solution. The immersive centrifugal/rotary jet spinning technique is similar to wet spinning, but the resulting fibers are collected on a rotary drum or deposited along the walls of a funnel. The most popular approaches to controlled microfluidic production of calcium alginate microfibers are using coaxial needles (where the central core carries an alginate solution, and the sheath flow is that of CaCl_2_ cross-linking solution) or similarly structured microfluidic chips [12,14].

In the calcium alginate microfiber production approaches discussed so far such as wet-spinning technology, immersive centrifugal rotary jet technology, and microfluidic spinning technology, the goal is to produce aligned fibers (such as in immersive centrifugal spinning and wet spinning) or to deposit fibers in a pre-designed pattern (via microfluidic spinning). We propose a variation of the traditional wet-spinning approach where the alginate fiber is not drawn through the cross-linking solution to obtain the straight fibers or rolled onto a drum. In the proposed immersed microfluidic spinning technology, the alginate fibers are injected through the needle into the cross-linking solution where the fiber can be either deposited in relatively straight segments or to produce a densely coiled stochastic fiber mesh. Parameters that determine the topology of the resulting calcium alginate fiber network are discussed below.

In our approach, we are using a single core needle attached to a syringe filled with an alginate solution. The syringe’s volumetric displacement is controlled by a pump. The needle of the syringe is immersed in a bath of CaCl_2_ solution. As the liquid jet of alginate is ejected from the needle, it is exposed to the cross-linking solution and becomes solidified.

This paper, investigates the influence of the needle size, the volumetric flow rate of the alginate solution, and the weight % concentration of the alginate solution on the diameter of the resulting calcium alginate microfiber.

The presented study of the immersed microfluidic spinning of the calcium alginate microfibers is instrumental for the design, fabrication, and optimization of the sacrificial microfiber network towards production of the advanced vascularized tissue engineered constructs.

## 2. Materials and Methods

Solutions of sodium alginate of three concentrations (2 wt%, 4 wt%, and 6 wt%) were prepared by dissolving alginic acid sodium salt (Sigma-Aldrich, St. Louis, MO, USA) in deionized (DI) water followed by stirring for 3 h at 60 °C. The 1 wt% calcium chloride solution was prepared by dissolving the calcium chloride powder (Sigma-Aldrich, MO, USA) into the DI water, followed by vortex mixing for 2 min.

**Figure 1:**
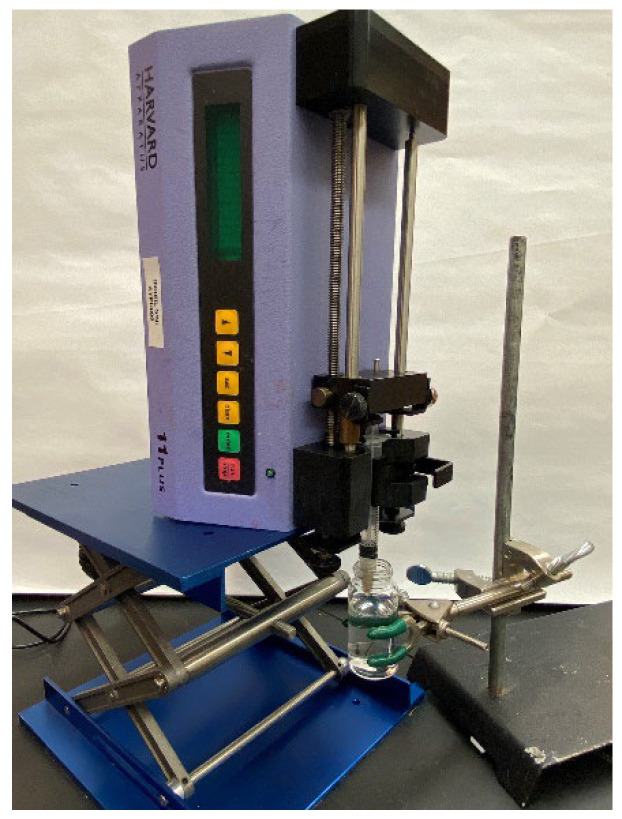
The immersed microfluidic spinning setup that includes a pump displacing the plunger of a syringe filled with a sodium alginate solution. The needle of the syringe is immersed in a bath of calcium chloride.

Figure 1 demonstrates the setup of the immersed microfluidic spinning. In this setup, a syringe pump (Harvard Apparatus, Holliston, MA, USA) is fitted with a 3 mL syringe (BD, NJ, USA) with an attached single core needle (Jensen Global Inc., Santa Barbara, CA, USA). Three different sets of needles with inner diameters of 30 g (810 microns), 25 g (455 microns), and 20 g (250 microns) were used to generate fibers. Three different volumetric flow rates of 1 mL/min, 2 mL/min, and 2.7 mL/min (maximum flow rate achievable by the pump) were utilized to study fabrication of the spun microfibers. The sodium alginate solution was ejected out of the needle that was immersed in a vial of 1 wt% calcium chloride cross-linking solution.

**Figure 2:**
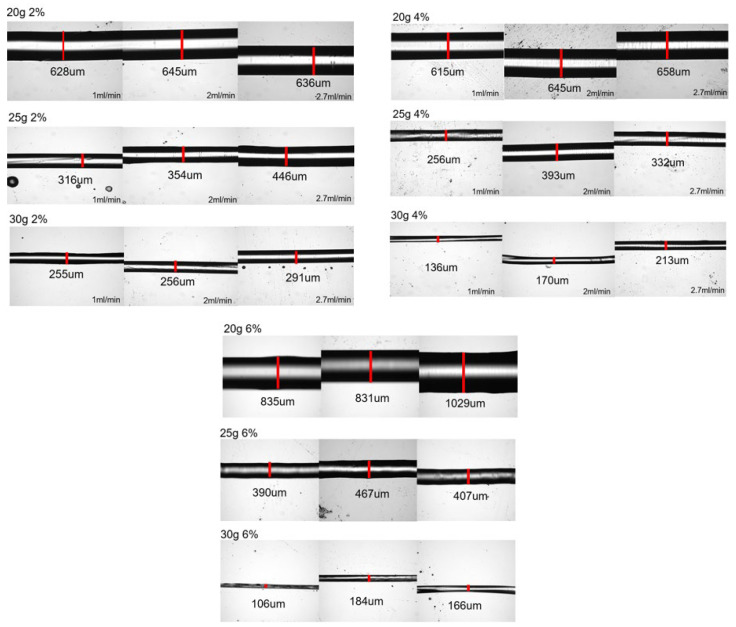
Optical pictures of the produced fibers. The images of the fibers are grouped into the sets of pictures of fibers made from sodium alginate of 2 wt%, 4 wt%, and 6 wt% where each row of images presents the results of spinning with a different needle gauge (of 20 g, 25 g, and 30 g, respectively). In each set of three sequential pictures, these images represent the increase in the volumetric flow rate from 1 mL/min to 2 mL/min to 2.7 mL/min. The white regions on the images of most of the fibers do not mean that these are hollow fibers but are indicative of the fact that for larger fibers, top sections of the fiber are not in focus.

The cross-linked fibers were collected from the vial with tweezers and studied under the high-power Nikon Eclipse microscope (Nikon Instruments Inc., Tokyo, Japan). The microfabricated gauge with a 100-micron gap was imaged next to the fibers (not pictured in Figure 2) to verify the fidelity of the measurements. The SPOT Basic Imaging software (Spot Imaging, Sterling Heights, MI, USA) was used to observe and measure the diameter of the fibers.

In order to study the dissolution rate of the fibers, 0.2 g of ethylenediaminetetraacetic acid (EDTA, Thermo Fisher Scientific, Waltham, MA, USA) and 0.3 g of disodium citrate (DSC, Sigma-Aldrich, St. Louis, MO, USA) were added to the solution of 50 mL of Dulbecco’s Modified Eagle Medium (DMEM, Thermo Fisher Scientific, Waltham, MA, USA). The fibers were prepared by the immersed fluidic spinning technique described above. The spun fibers were then extracted by tweezers from the calcium chloride cross-linking solution. Then, a 1 mm long piece of fiber was covered by the hydrogel. In order to avoid studying the parts of the fiber distorted by the handling, the ends of the longer piece of fiber were cut off from the ends where the tweezers compressed the fibers and we studied the length of the fiber not affected by the handling process.

The gelatin methacryloyl (GelMA) hydrogel was synthesized by dissolving type A gelatin from porcine skin (Sigma-Aldrich, St. Louis, MO, USA) at 10% (*w*/*v*) in Dulbecco’s phosphate-buffered saline (DPBS) (Thermo Fisher Scientific, Waltham, MA, USA) and stirring at 50 °C for one hour. Methacrylic acid (Sigma-Aldrich, St. Louis, MO, USA) was added to the solution at 10% (*v*/*v*) and allowed to react for one hour. The reaction was finished by adding 5x volumes of DPBS. The resulting solution was dialyzed for 7 days with distilled water at 37 °C to remove unreacted methacrylic anhydride using dialysis membranes; the water was changed twice daily. The resulting solution was frozen and lyophilized for 5 days and stored at −80 °C for future use. Lyophilized GelMA was dissolved at 5% (*w*/*v*) in DPBS at 70 °C in a water bath containing 0.2% (*w*/*v*) lithium phenyl-2, 4, 6-trimethylbenzoylphosphinate (LAP) (Allevi, Inc., Philadelphia, PA, USA) as the photoinitiator.

The fiber was embedded in the hydrogel and then exposed to UV light (405 nm) to crosslink the GelMA hydrogel. The hydrogel sample with embedded fiber was then covered by the prepared DMEM/EDTA/DSC solution for fiber dissolution. Visual observation of the fiber under the microscope was performed periodically to record when the fiber was completely dissolved, and the hollow channel was left in the hydrogel.

## 3. Results and Discussion

### 3.1. Dependence of the Fiber Diameter on Sodium Alginate Flow Rate, Alginate wt%, and Needle Gauge

Figure 2 presents the images of fibers generated by the immersed microfluidic spinning, while Table 1 summarizes the average fiber diameters for each set of parameters.

**Figure 3:**
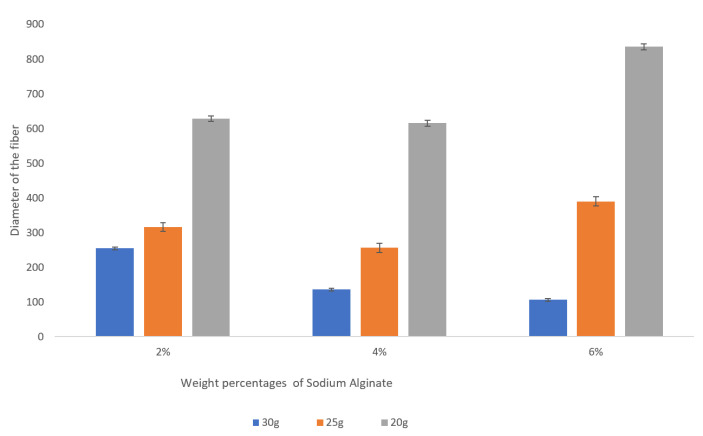
Dependence of the fiber diameter on the gauge of the needles for various concentrations of sodium alginate with the sodium alginate flow rate kept at 1 mL/min. The error bars represent the standard deviation of the measured diameters.

**Figure 4:**
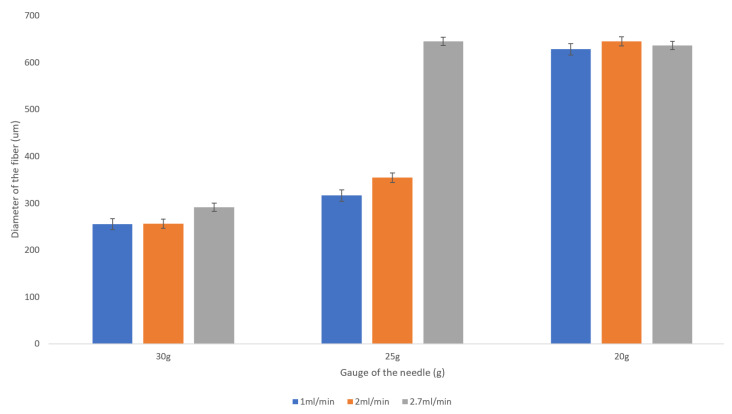
Dependence of the fiber diameter on the flow rate of the 2 wt% sodium alginate. There is no pronounced trend for the resulting fiber diameter with the increase in the flow rate from 1 mL/min to 2.7 mL/min. The error bars represent the standard deviation of the measurements.

**Figure 5:**
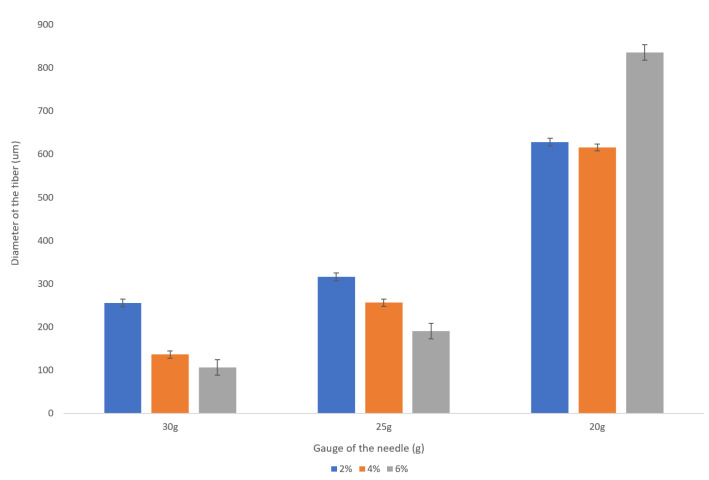
Dependence of the fiber diameter on the concentration of the sodium alginate with the flow rate of 1 mL/min. The error bars represent the standard deviation of the measurements.

It is clear from the observed data that the gauge of the needle plays a very significant role in the resulting diameter of the spun microfiber—the smaller the inner diameter of the needle (the greater its gauge)—the smaller is the resulting fiber diameter. Figure 3 illustrates the dependence of the fiber diameter on the gauge of the needle. In order to demonstrate the trend, we selected a specific volumetric flow rate of the calcium alginate of 1 mL/min. We can see that for all concentrations of sodium alginate, the use of the smaller diameter needles (larger gauge needles) results in smaller diameter calcium alginate fibers. The fiber diameters given in Figure 3, Figure 4 and Figure 5, as well as in Table 1, are the mean diameters measured using three sets of data and the error bars represent the measured standard deviation.

It is important to observe that the fiber diameters are smaller than the inner diameters of the needles. The physical causes of this phenomenon will be considered in Section 3.3.

The flow rate of the sodium alginate does not noticeably affect the resulting fiber diameter as evidenced by the plot presented in Figure 4.

We observed a significant decrease in a fiber diameter produced with a 30 g needle as sodium alginate concentration is increased from 2 wt% to 6 wt%. For example, for the given flow rate of 1 mL/min, the fiber diameter is reduced from about 250 microns to about 100 microns (see Figure 5). This is a promising trend that should be studied more closely given the fact that the increase in the concentration of the sodium alginate solution does not always result in smaller diameter fibers when the larger inner diameter needles (20 g, 25 g) are used.

### 3.2. Topology of the Deposited Microfluidically Spun Fiber Network

**Figure 6:**
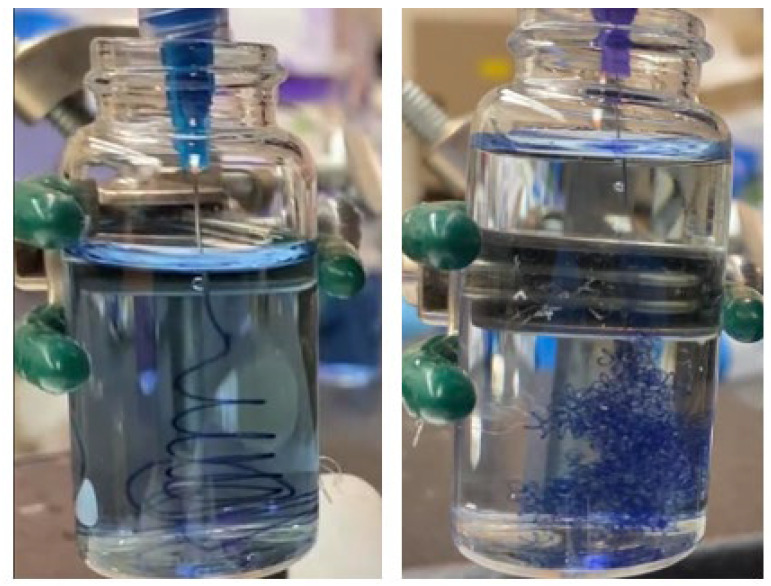
The observed topology of the fiber deposited via immersed microfluidic spinning. In both cases 1 mL/min volumetric flow rate of the sodium alginate was used. The left picture represents spinning of 6 wt% sodium alginate with a 20-gage needle and the right picture presents the spinning of 4 wt% sodium alginate with 25-gage needle.

For many applications, including tissue engineering, the topology of the deposited fiber could play an important role. We have observed (see Figure 6) that for the same flow rate (1 mL/min), a higher concentration (6 wt%) of sodium alginate produces larger loops of the deposited fiber, while the smaller (4 wt%) concentration of sodium alginate produces tighter loops of fiber, more appropriate for higher density of the deposited fibers. The blue food dye was added to the sodium alginate solution to facilitate the observation of the deposited fiber networks. Videos S1 and S2 of the immersed microfluidic spinning are provided as the Supplementary Materials.

While we have not characterized the exact topology of the resulting microfluidically spun alginate fiber networks, it is clear that the topology of the deposited networks can be optimized (based on the application) by varying the concentration of the alginate solution and other parameters. We believe that a higher concentration of the alginate solution results in denser solution with higher inertia, explaining the observed trajectory of the jetted fiber.

Once the fiber network is produced, the cross-linking solution can be drained or aspirated, and the cell-laden hydrogel can be added to the fiber network. If the whole fiber network is desired to be transferred to a separate location once it is created, then the deposition can be conducted within the plastic or metallic mesh inserted inside the glass beaker. Thus, after the generation of the fiber network, that mesh can be lifted from the glass beaker, draining the cross-linking solution, and the fiber network can be transferred to a different volume (see Figure S1 in the Supplementary Materials).

We expect that due to the dynamic nature of the deposition of the fiber network and stretching of the fibers as they fall into the cross-linking solution, there will be some variation in fiber diameter (see Table 1 for the standard deviations of the diameter of the produced fibers). Therefore, if precise control of the fiber diameter is desired, then such spinning techniques as wet spinning or microfluidic spinning surveyed in Section 1 will be a more appropriate fabrication approach.

### 3.3. Deposited Fiber Diameter Is Smaller than the Inner Diameter of the Needle

The narrowing of the fluid jet issuing from the needle into another fluid can be observed in many applications, including in water flowing from the kitchen faucet. The observed phenomenon relates to the acceleration on the falling jet after it issues from the constraining pipe. As continuity of the jet is preserved and the same volume of the jet now goes through a larger distance under the gravitational acceleration, the narrowing of the jet is observed.

The combination of the continuity equation and the Navier–Stokes equation in the falling jet constrained by the surface tension can be described by the modified Bernoulli Equation [16] in the form of Equation (1):

(1) $$P+\frac{\alpha }{2}\rho \upsilon^{2}+\rho gz+\gamma \left(\frac{\partial A}{\partial V}\right)+\omega_{los}=const.$$

where *P* is the applied pressure and *υ* is the mean velocity of the falling jet, *g* is the gravitational acceleration, *z* is the distance from the tip of the needle, *ρ* is the density of the falling jet, *γ* is the coefficient of the surface tension, *ω*_los_ is the dissipation energy due to viscous resistance, *A* is the cross-sectional area of the jet at a specific coordinate z, dV is the infinitesimal volume of the fluid element, and *α* is the coefficient describing the velocity profile distribution within the jet, with alpha of 1 representing the uniform velocity and alpha of 2 representing the parabolic velocity profile.

This analysis was successfully performed for the falling water jet in the air [16]. The analogous analysis might not yield the satisfactory results due to the presence of the fluid significantly more viscous than air that consequently will absorb the significant portion of the energy of the falling jet. However, the main underlying physical principles remain valid, and they explain the narrowing of the jet issuing from the needle during the immersed microfluidic spinning.

### 3.4. Dissolution of the Fluidically Spun Microfibers

**Figure 7:**
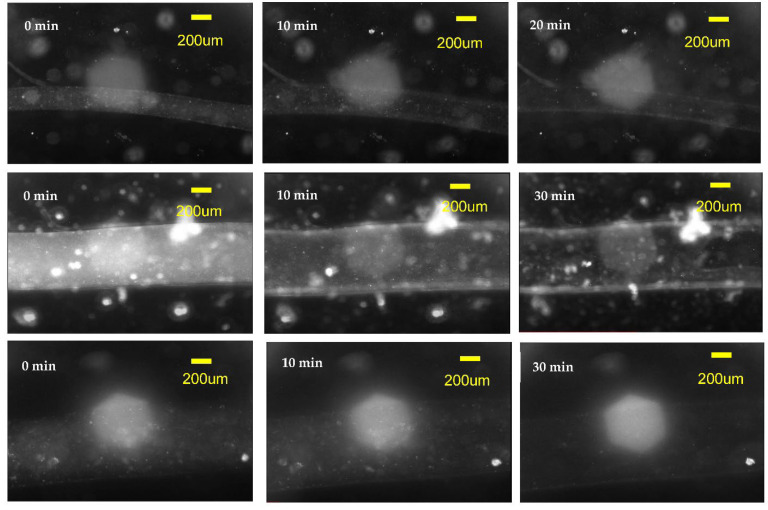
The dissolution of 8% alginate fibers produced with the immersed microfluidic spinning. Top row of images is a timed sequence of the dissolution of the 240-micron diameter fiber, the middle row contains images of the dissolution of the 680-micron fiber, while the bottom row contains images of the 800-micron diameter fiber. All fibers had a length of 1 mm and were covered by a layer of hydrogel. The DMEM/EDTA/DSC solution was used to dissolve the calcium alginate fibers. The dissolution was observed in the microscope and a timed sequence of images was taken. The initial images before dissolution are on the left, the middle images correspond approximately to 10 min after the dissolution started. The leftmost images are the images that did not change with time (i.e., the fiber dissolution was completed)—for the 240-micron fiber it took 20 min to dissolve, while for 680- and 800-micron fibers it took around 30 min to dissolve.

The segments of 1 mm fibers of three diameters—240 μm, 680 μm, and 800 μm—spun from 8% alginate were covered by hydrogel followed by immersion into the DMEM/EDTA/DSC solution as described in Section 2. It is difficult to observe the fiber dissolution process since both the calcium alginate fiber and the hydrogel matrix into which the fiber is embedded are soft transparent media. We discovered that observation of nano inclusions and/or tiny bubbles of the air trapped inside the alginate fibers is the most convenient way to discover the process of fiber dissolution. When the fiber dissolves, these inclusions can move and are washed away. The process of elimination of the inclusions as the fiber is dissolved can be followed in observing the timed sequence of images of the fiber segments presented in Figure 7. Images before the dissolution of fibers are presented in the left column of images and the middle column of images were taken after 10 min of immersing the fibers in the dissolving solution. The images in the rightmost column were taken after 20 min for 240-micron fibers and after 30 min for 680-micron and 800-micron fibers when there was no longer a change in appearance of the fiber and we could conclude that the dissolution process was complete. Therefore, we can conclude that the 800- and 680-micron fibers were dissolved in about 30 min, while the 240-micron diameter fiber segment was dissolved in about 20 min.

## 4. Conclusions

A simple and robust method of immersed microfluidic spinning capable of generating calcium alginate fibers with diameters between 100 microns and 1000 microns is reported. A series of experiments were performed where the influence of three parameters—the volumetric flow rate of sodium alginate (1 mL/min, 2 mL/min, and 2.7 mL/min), the concentration of sodium alginate (2 wt%, 4 wt%, and 6 wt%), and the inner diameter of the needle (20 g, 25 g, and 30 g) were studied and their influence on the resulting calcium alginate fiber diameter was reported. The study has demonstrated that the flow rate of the alginate does not significantly affect the resulting diameter of the fibers, that the reduction in the size of the needle decreased the resulting diameter of the fibers, and that the increase in the sodium alginate concentration decreases the fiber diameter for a 30 g needle, but there is no pronounced effect for larger needles. The topology of the deposited calcium alginate fiber network depends on the concentration of the sodium alginate, with tighter, denser coils of fibers being deposited when the smaller wt% of sodium alginate was used. These fibers can be dissolved in DMEM/EDTA/DSC solution in 20 to 30 min (depending on the fiber diameter), leaving behind the hollow channels in the hydrogel matrix.

The immersed microfluidic spinning technology generating dissolvable calcium alginate microfibers with a diameter of several hundred microns that can be deposited in tight, small-coiled stochastic networks can be useful for creation of vascularized tissue constructs. Our future research will include a detailed study of the cell-laden hydrogels with an embedded sacrificial fiber network produced with the immersed fluidic spinning. Live/dead cell viability studies will be conducted to ensure that the cells will survive in DMEM/EDTA/DSC solution during the time required for dissolution of the calcium alginate fibers.

## Figures and Tables

**Table 1:** Mean values and standard deviations of diameters of the cross-linked calcium alginate fibers generated via immersed microfluidic spinning using different volumetric flow rates of sodium alginate, different concentrations of sodium alginate solution, and the gauges of the needles.

Needle Size	Diameter of Fiber (μm)	Needle Size	Diameter of Fiber (μm)	Needle Size	Diameter of Fiber (μm)
**2% alginate**					
1 mL/min		2 mL/min		2.7 mL/min	
30 g	255 ± 4	30 g	256 ± 20	30 g	291 ± 13
25 g	316 ± 13	25 g	354 ± 13	25 g	446 ± 8
20 g	628 ± 8	20 g	645 ± 13	20 g	636 ± 8
**4% alginate**					
1 mL/min		2 mL/min		2.7 mL/min	
30 g	136 ± 19	30 g	170 ± 30	30 g	213 ± 66
25 g	256 ± 2	25 g	393 ± 15	25 g	332 ± 2
20 g	615 ± 22	20 g	645 ± 7	20 g	658 ± 8
**6% alginate**					
1 mL/min		2 mL/min		2.7 mL/min	
30 g	106 ± 3	30 g	184 ± 9	30 g	166 ± 3
25 g	390 ± 12	25 g	467 ± 8	25 g	407 ± 8
20 g	835 ± 26	20 g	831 ± 17	20 g	1029 ± 15

## Data Availability

The data presented in this study are available on request from the corresponding author.

## Supplementary Materials

The following supporting information can be downloaded at: https://www.mdpi.com/article/10.3390/mi14020318/s1. Figure S1: The proposed process for producing and transporting the fiber network with the immersed microfluidic spinning. The meshed cage (a) is inserted inside the beaker with the calcium alginate cross-linking solution (b) followed by the immersed microfluidic spinning of the fiber inside the meshed cage (c) and removal of the cage with the produced fiber network (d) that is now ready for transport to a separate location. Video S1: Fluidic Spinning 6 per 20 g 1 mL/min, Video S2: Fluidic Spinning 4 per 25 g 1 mL/min.
