# Supplementary material for: Dissolvable Calcium Alginate Microfibers Produced via Immersed Microfluidic Spinning
Source: Micromachines (Basel). 2023 Jan 26;14(2):318. doi: 10.3390/mi14020318 (PMC9965352; doi:10.3390/mi14020318)
Supplement: Supplementary file 1 [file micromachines-14-00318-s001.zip › micromachines-2145348-supplementary-for final/Supplementary Materials Figure S1.pdf]

Supplementary Materials

for

"Dissolvable Calcium Alginate Microfibers produced via Immersed Microfluidic Spinning"

by Tuo Zhou, Sahar NajafiKhoshnoo, Rahim Esfandyarpour, and Lawrence Kulinsky

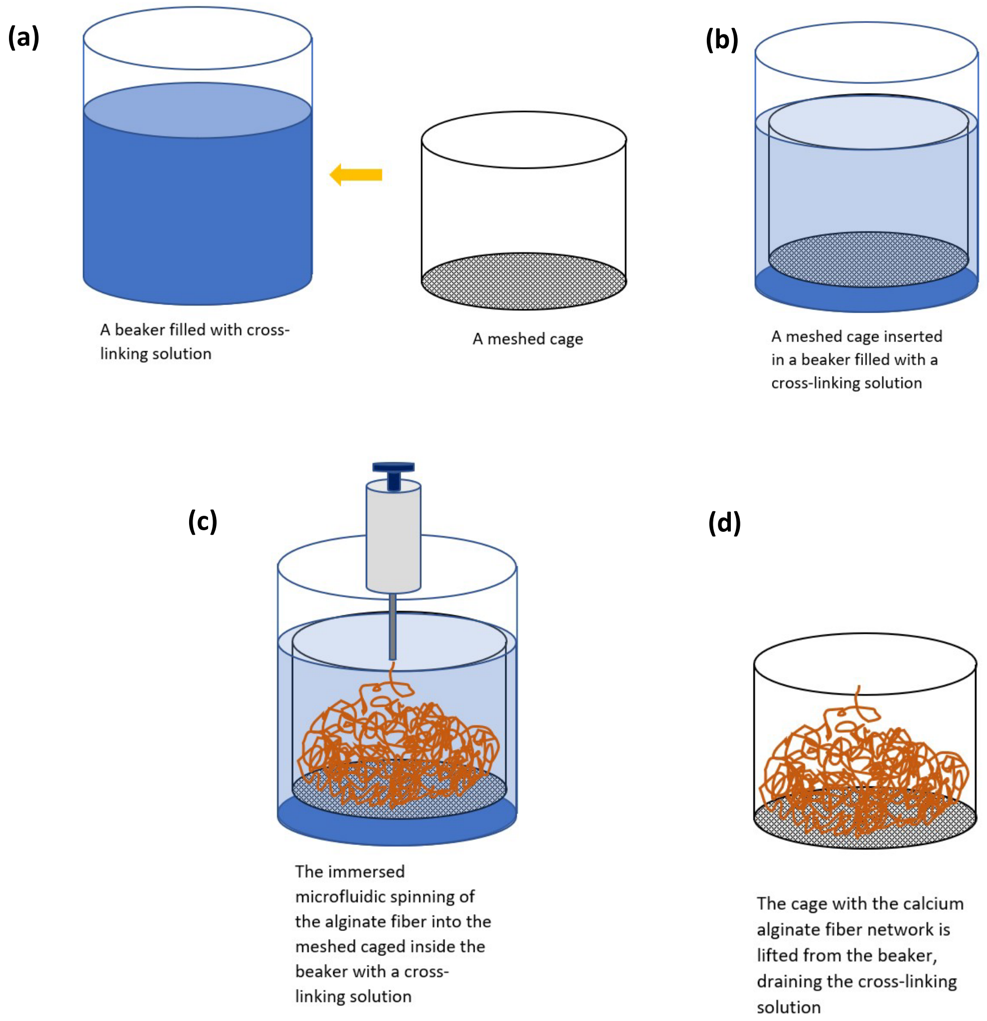

Figure S1. The proposed process for producing and transporting the fiber network with the immersed microfluidic spinning. The meshed cage (a) is inserted inside the beaker with the calcium alginate cross-linking solution (b) followed by the immersed microfluidic spinning of the fiber inside the meshed cage (c) and removal of the cage with the produced fiber network (d) that is now ready for transport to a separate location
